# An epigenetic clock for gestational age based on human umbilical vein endothelial cells from a diverse population of newborns

**DOI:** 10.21203/rs.3.rs-3112428/v1

**Published:** 2023-06-30

**Authors:** Gang Peng, David W. Sosnowski, Susan K. Murphy, Sara B. Johnson, David Skaar, William S. Schleif, Raquel G. Hernandez, Hector Monforte, Hongyu Zhao, Cathrine Hoyo

**Affiliations:** Indiana University School of Medicine; Johns Hopkins Bloomberg School of public Health; Duke University Medical Center; Johns Hopkins Bloomberg School of public Health; North Carolina State University; Johns Hopkins School of Medicine; Johns Hopkins School of Medicine; Johns Hopkins All Children’s Hospital; Yale University School of public Health; North Carolina State University

**Keywords:** DNA methylation, race/ethnicity, aging, newborn, human umbilical vein endothelial cells

## Abstract

**Background:**

Epigenetic clocks are emerging as a useful tool in many areas of research. Many epigenetic clocks have been developed for adults; however, there are fewer clocks focused on newborns and most are trained using blood from European ancestry populations. In this study, we built an epigenetic clock based on primary human umbilical vein endothelial cells from a racially and ethnically diverse population.

**Results:**

Using human umbilical vein endothelial cell [HUVEC]-derived DNA, we calculated epigenetic gestational age using 83 CpG sites selected through elastic net regression. In this study with newborns from different racial/ethnic identities, epigenetic gestational age and clinical gestational age were more highly correlated (r = 0.85), than epigenetic clocks built from adult and other pediatric populations. The correlation was also higher than clocks based on blood samples from newborns with European ancestry. We also found that birth weight was positively associated with epigenetic gestational age acceleration (EGAA), while NICU admission was associated with lower EGAA. Newborns self-identified as Hispanic or non-Hispanic Black had lower EGAA than self-identified as non-Hispanic White.

**Conclusions:**

Epigenetic gestational age can be used to estimate clinical gestational age and may help index neonatal development. Caution should be exercised when using epigenetic clocks built from adults with children, especially newborns. We highlight the importance of cell type-specific epigenetic clocks and general pan tissue epigenetic clocks derived from a large racially and ethnically diverse population.

## Background

Over the past decade, epigenetic clocks have evolved as promising indicators of health, aging, and age-related diseases in adults [1–3]. These clocks allow researchers to move beyond the analysis of locus-specific methylation to understanding broader functional implications for the epigenome, and subsequent health outcomes, in response to stress exposure. This is particularly valuable early in the life course when exposure to stressors is hypothesized to influence developmental trajectories and epigenetic processes may underlie these changes over time. In part to address concerns about accuracy of adult-based clocks in younger populations [4, 5], several epigenetic clocks have been developed in pediatric samples [4, 6–8]. However, these clocks are limited by their development using different, individual tissue and cell types (i.e., cord blood, buccal cells) and their reliance on samples primarily from individuals of European ancestry [7, 8]. Thus, it remains unclear how well existing clocks perform across different tissue or cell populations, or within more racially and ethnically diverse samples.

One of the most common pediatric epigenetic clocks is the PedBE clock [4]. This clock, consisting of 94 CpG sites, was generated using samples from more than 1,000 participants aged 0–20 years and was trained using buccal cells, which are a desirable tissue source in the pediatric population given the non-invasive nature of tissue collection. This clock demonstrates a strong, positive correlation with chronological age (*r* = .98) and accuracy over time. In addition, deviations in PedBE age from chronological age are linked to obstetric (e.g., length of gestation) and developmental outcomes (e.g., autism spectrum disorder). However, due to limited data availability, the research team could not account for race or ethnicity when training and testing the PedBE clock [4]. Thus, the performance of this clock in racially and ethnically diverse populations is unclear. It is also unknown whether it is similarly associated with obstetric and developmental outcomes across racial and ethnic groups.

Although the PedBE clock was developed to account for the greater DNAm changes in childhood as compared to adulthood [9], it is based on from participants aged from 0–20 years, and may face similar challenges as do adult clocks when applied to newborns. Indeed, several pediatric epigenetic clocks were created to investigate epigenetic gestational age at birth (as opposed to any time after birth). Specifically, four clocks were created solely in neonatal samples: Knight et al [6], Bohlin et al [7], Falick Michaeli et al [10], and Haftorn et al (EPIC GA) [8]. The samples used to generate each clock were mostly racially and ethnically homogeneous, with Bohlin et al and Haftorn et al sampling Norwegian infants, and Falick Michaeli et al. sampling Israeli infants. Although Knight et al. had a more racially admixed sample, the training data included only about 17% infants of African ancestry. Each of the study samples used to generate neonatal clocks relied on DNA derived from mixed cord blood leukocytes, though there was variation in platforms used to assess methylation. Knight et al used methylation data from the Illumina 27K and 450K arrays, Bohlin et al used data solely from the 450K array, Haftorn et al used data solely from the EPIC array, and Falick Michaeli et al used reduced representation bisulfite sequencing (RRBS) to construct their neonatal epigenetic clocks. Due to these and other study-specific factors, zero-order correlations between epigenetic age and gestational age vary among the clocks, ranging from 0.71 to 0.99. Interestingly, only Knight et al examined whether their clock was associated with obstetric outcomes; the authors found that epigenetic age acceleration was positively associated with infant birthweight (independent of gestational age), after adjusting for infant sex and genetic ancestry [6].

As evidenced in the existing literature, numerous epigenetic clocks have been generated. These clocks perform reasonably well in the pediatric population and are associated with relevant obstetric and developmental outcomes. However, these clocks, specifically those aimed at estimating gestational age, are limited by their use of DNA derived from a single tissue source (mixed leukocytes from cord blood) and reliance on samples of mostly European ancestry. Moreover, it is unclear how social stressors experienced by the mother, which may vary based on racial identity and related experiences [11], contribute to epigenetic gestational age. Extant data supports conflicting evidence for the relationship between stress exposure and epigenetic age deviation (i.e., acceleration and deceleration) in pediatric populations [12], but no studies have examined whether a mother’s exposure to social adversity prior to the index pregnancy, a potentially potent indicator of maternal social risk, is associated with infant epigenetic gestational age. Thus, the goals of the present study were to (1) use a racially and ethnically diverse sample of infants to generate a new epigenetic gestational age clock with DNA derived from a unique cell population (i.e., human umbilical vein endothelial cells [HUVEC]); and (2) examine whether obstetric outcomes and maternal stressors before and during the pregnancy were associated with deviations in this pediatric epigenetic clock.

## Results

### Epigenetic Gestational Age

**Figure 1:**
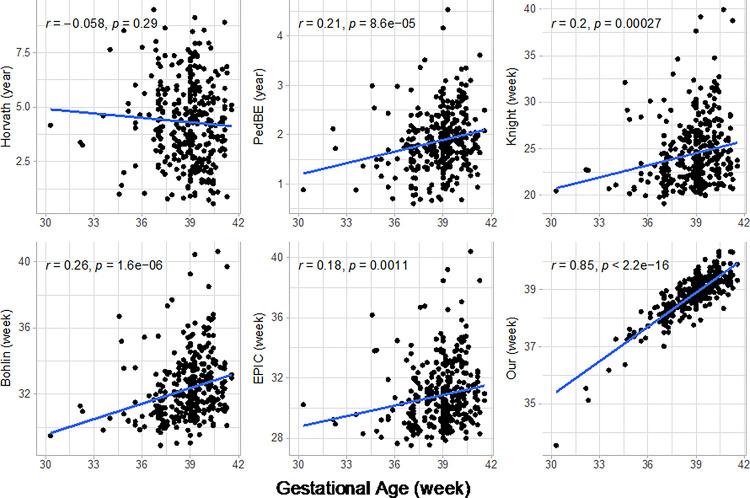
Correlation between epigenetic gestational age and clinical gestational age. (r: Pearson correlation coe cient, p: p-value.)

We built a clock using data derived from HUVEC-derived DNA from a diverse population (Table 1) and 83 CpG sites were selected by elastic net with 10-fold cross validation (**Supplementary Table 1**). Epigenetic gestational age was also estimated using five existing clocks. There was little overlap among the CpG sites used in the six clocks (**Supplementary Fig. 1**). Although both EPIC [8] and Bohlin’s [7] clocks were created from The Norwegian Mother and Child Cohort Study (MoBa), only 11 CpGs were common between the two clocks. Figure 1 shows the correlation between epigenetic gestational age across the six clocks and gestational age at delivery from obstetric records. Only our clock demonstrated a very high correlation with clinical gestational age. While Pearson correlation coefficients ranged from r=−0.06 to 0.26 for other clocks, the correlation coefficient for our clock was r = 0.85. There was no significant association between Horvath’s clock [13] and clinical gestational age. This is likely due to Horvath’s clock being trained on adult samples, whereas the other five clocks were trained exclusively on pediatric populations or newborns [14]. Thus, we removed Horvath’s clock from subsequent analyses.

**Figure 2:**
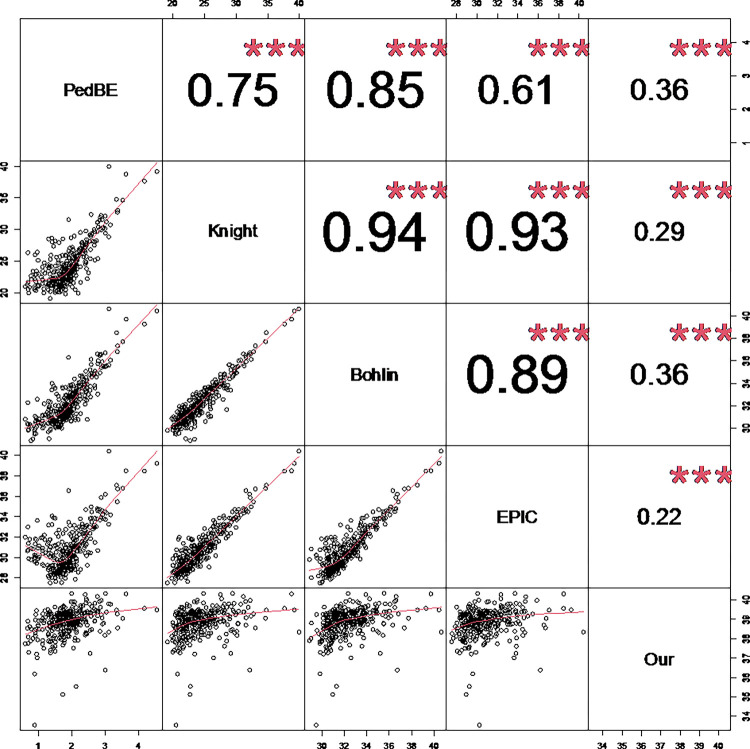
Correlation between five methylation clocks. Panels on the bottom left are scatterplots comparing paired methylation clocks. The numbers on the top right are Pearson correlation coefficients between paired methylation clocks. (*** p-value < 0.001.)

Correlations among the five remaining clocks were also compared (Fig. 2). Knight’s clock [6], Bohlin’s clock, and the EPIC GA clock were very highly correlated (r > 0.89). There were moderate correlations between PedBE [4] and each of the three clocks (r = 0.61 to 0.85). Epigenetic gestational age estimated from our clock was distinct from the other clocks (r of approximately 0.3).

To probe the association between racial/ethnic identity and newborn epigenetic age, we divided our data into two groups: individuals who identified as Black (including non-Hispanic Black and Hispanic Black) and those who identified as non-Black (including non-Black Hispanic, non-Hispanic White, and individuals of other identities). We found that the correlation between epigenetic age and epigenetic gestational age was much lower among newborns born to mothers who identified as Black compared to those who did not for Knight’s clock (−18.21%), Bohlin’s clock (−21.28%), and the EPIC GA clock (−39.07%). In contrast, the correlation was similar between Black and non-Black subgroups for the PedBE clock (1.51%) and our clock (1.52%) (**Supplementary Table 2**).

### Association between Epigenetic Gestational Age Acceleration, Maternal Social Adversity, and Perinatal Covariates

**Figure 3:**
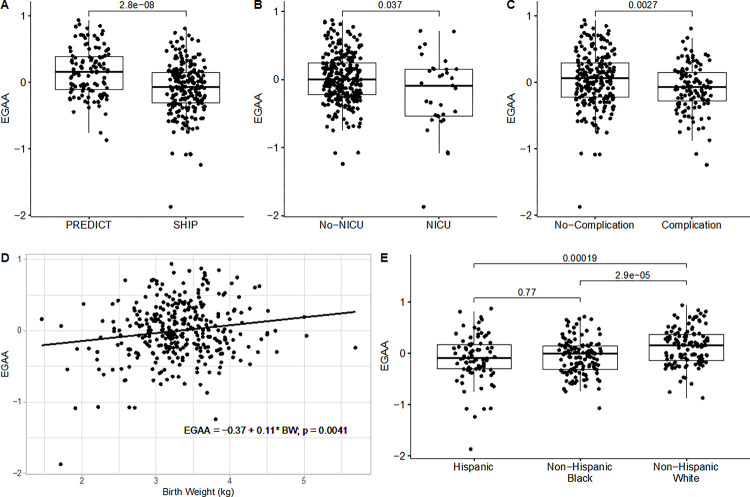
Covariates significantly associated with epigenetic gestational age acceleration (EGAA). (A) Cohort; (B) NICU status; (C) Pregnancy complications; (D) Birth Weight; (E) Race/Ethnicity. T-tests were used for categorical data and the numbers between groups are p values comparing noted groups. Linear regression was used to evaluate relationships between EGAA and other continuous covariates. The grey area in panel D shows the 95% confidence interval. NICU: neonatal intensive care unit; BW: birth weight; p: p-value.

We found that only the epigenetic age calculated from our clock demonstrated a high correlation with clinical gestational age. Thus, we calculated Epigenetic Gestational Age Acceleration (EGAA), the residual of the linear regression of epigenetic gestational age on clinical gestational age, from our clock and estimated the association between EGAA and social adversity scores that included maternal exposure to adverse childhood experiences, financial stress, social support, discrimination, depression and perceived stress, and other perinatal covariates. Notably, no significant relationship was found between EGAA and social adversity scores (**Supplementary Table 3**). Only five covariates were significantly associated with EGAA (Fig. 3): in the SHIP cohort, newborns with neonatal health concerns, and those whose mother had a pregnancy complication had slower EGAA, whereas newborns born to non-Hispanic White women, and those with larger birth weights showed faster EGAA.

## Discussion

We applied six clocks, including one we developed, to HUVEC-derived DNA methylation data collected from a racially and ethnically diverse sample of newborns. Only our clock was highly correlated with clinical gestational age; Horvath’s clock, a cornerstone of epigenetic aging research, in contrast, was not significantly associated with clinical gestational age in newborns (Fig. 1). Because of limit of sample size, we cannot build an epigenetic gestational age clock from an independent dataset first and then validate the clock on our data. The correlation coefficient from our clock shown in Fig. 1 should be overestimated. Knight’s clock was built on a dataset with 207 newborns. The correlation is 0.99 in the training dataset and 0.91 (8% lower) in the testing dataset [6]. EPIC GA clock was built on a dataset of 755 newborns. The correlation is 0.84 in the training set and 0.71 (15% lower) in the testing set from another country that might explained the large difference [8]. Our clock was trained from a data set of 336 newborns. The correlation is 0.85 in the training set. Considering the high diversity of our data, we can still get a correlation of 0.6 if correlation is 30% lower (doubled of EPIC GA clock) in the testing set. The correlation is still much higher than the other clocks.

One reason clocks may perform poorly in newborns is that they are often trained on data from children (PedBE) or adults (Horvath’s clock). However, Knight’s clock, Bohlin’s clock, and the EPIC GA clock were each developed using newborn training datasets; nonetheless, epigenetic age estimated from these three clocks still demonstrated much lower correlations with clinical gestational age in our dataset than in the original investigators’ own testing datasets [6–8]. There are at least two possible reasons for this. First, the three existing neonatal clocks were built with methylation data derived from cord blood or blood spots, whereas DNA samples in our study were derived from primary HUVECs using a validated methodology. Epigenetic clocks trained using data from one cell type may have poorer performance in other cell types [4, 15, 16]. Second, the racial/ethnic composition of the training datasets may also play a role. Only 17% of training samples were from Black participants for Knight’s clock and both Bohlin’s clock and the EPIC GA clock were based on The Norwegian Mother and Child Cohort Study (MoBa), which is primarily comprised of White participants. Our sample was more racially and ethnically heterogeneous: 35.4% identified as non-Hispanic White, 38.7% identified as non-Hispanic Black and 24.7% identified as Hispanic (Table 1). Social experiences that covary with racial/ethnic identity may be associated with methylation profiles and estimates of epigenetic age [2, 17, 18]. In our own data, we saw methylation differences between infants born to mothers who identified as Black as compared those who did not, suggesting the possibility that racialized experiences may underlie the observed associations (**Supplementary Fig. 2**).

In contrast to the Bohlin, Knight and EPIC GA clocks, the PedBE clock was developed from samples collected from individuals 0 to 20 years old. Nonetheless, the correlation between PedBE and our clock was similar to the correlation between the other three newborn clocks and our clock. This might be explained by differences in tissue type. PedBE was constructed from buccal cells, which, like the HUVECs used to generate our data, are a type of epithelial cell.

Knight’s clock, Bohlin’s clock, and EPIC GA clock were constructed from majority European ancestry populations, therefore, they may be expected to have poorer performance in more racially and ethnically diverse populations. Although the racial/ethnicity composition of the training dataset for the PedBE is not published, the correlations we observed suggest that the training data may have from a more diverse population than the Knight, Bohlin and EPIC GA clocks (**Supplementary Table 2**).

We also found that EGAA was associated with birthweight and certain maternal demographic characteristics. Specifically, the mean increase in EGAA was 0.11 (95% CI: 0.04–0.19) weeks per kg at birth. This estimate is similar to Bright et al.’s estimation [19]. We did not see significant associations with maternal social adversity in this study, however. Social adversity scores varied by cohort and race/ethnicity (**Supplementary Fig. 3**); thus, we may need a larger cohort to see the association in a stratified analysis.

Consistent with prior studies, estimates of EGAA from our clock varied by racial/ethnic identity. Horvath proposed intrinsic epigenetic age acceleration (IEAA) that “measures ‘pure’ epigenetic aging effects that are not confounded by differences in blood cell counts” [2]. HUVECs are pure endothelial cells, so EGAA estimated from HUVECs should be similar to IEAA without confounding of differences in cell counts of the contribution cells. Indeed, prior evidence has found that Hispanic adults have lower intrinsic epigenetic aging than White adults. This is consistent with our findings in newborns (Fig. 3E). We also found that newborns identified as Black had lower EGAA, whereas Horvath found that individuals with African ancestry did not have lower IEAA. Newborns identified as Black were found to have lower birth weights even at the same clinical gestational age [20]. Birth weight of newborns identified as Black were significantly lower than those identified as Hispanic or White in our data (**Supplementary Fig. 4**). Lower EGAA among newborns identified as Black might be related to lower birth weights since we observed that lower birth weight was associated with lower EGAA (Fig. 3D). These race/ethnic differences, which may reflect differences in social experiences, including racialized experiences, were also found in our subcohorts, one of which was predominantly non-Hispanic Black, while the other was predominantly White (Table 1, Fig. 3A). Mothers who identified as Hispanic were more likely to have a pregnancy complication (54.2%) than those who identified as non-Hispanic (28.9%) in our data. Newborns who were transferred to NICU for additional care after birth were also more likely to have lower birth weights (**Supplementary Fig. 5**). These six significant variables were highly correlated. To control for the confounding effects of these variables, we used a multivariable linear regression between EGAA and the significant variables found in the unadjusted analysis. Four out of the six variables were significantly associated with EGAA (all except presence of a pregnancy complication and Non-Hispanic Black racial/ethnic identity) (**Supplementary Table 4**).

Some variables that capture pregnancy and neonatal health risks like NICU admission and pregnancy complications were associated with lower EGAA. Complications, NICU admission, and low birth weight have previously been associated with higher risk of developmental delay [21–23], while high birth weight has been associated with earlier onset of puberty [24]. Unlike the assumption that a lower epigenetic age in adults equates to better health, epigenetic age in pediatric samples may be ideal when concordant with chronological age – neither fast nor slow epigenetic aging is likely to be beneficial during early development. Thus, epigenetic gestational age could provide insight into the developmental stage of newborns.

The results of this study should be considered in light of several limitations. First, the population in this study was heterogeneous, including two cohorts and multiple race/ethnicity groups. Although this enhances external validity, it will be important to validate our findings in other samples. Second, our sample size was small, especially for preterm newborns, so may have been underpowered to detect a relationship between EGAA and social adversity. Third, while we addressed confounding analytically, residual and unmeasured confounding are still potential issues. Fourth, while our cell type, HUVEC, was novel and extends prior literature, we did not examine the utility of the clock in other cell populations. Finally, we did not examine clinical or behavioral outcomes beyond the neonatal period, which limits insight into whether EGAA is adaptative or non-adaptive.

## Conclusion

In summary, we developed an epigenetic clock from HUVECs; we found that it performed better than other epigenetic clocks of gestational age in our racially and ethnically diverse sample. Birth weight was positively associated with EGAA, and newborns identified as non-Hispanic Black and Hispanic had lower EGAA than those identified as White non-Hispanic. Our results suggest that caution should be exercised in applying epigenetic clocks built for adults to children, especially newborns. Both cell type and patterns of social experience associated with self-reported racial identity may be linked to changes in methylation profiles. Similar caution should be exercised when using epigenetic clocks built from specific tissue types or populations. To maximize applicability, it is important to build cell type-specific epigenetic clocks or, alternatively, to use pan tissue samples from a large racially and ethnically diverse population to build a general epigenetic clock. Lastly, epigenetic gestational age may provide insight into neonatal development; however, clinical data during infancy will help clarify its utility.

## Methods

### Study Participants

Pregnant women were enrolled between April 2018 and March 2020 in two cohorts—the Stress and Health in Pregnancy (SHIP) cohort in North Carolina and the Prospective Research on Early Determinants of Illness and Children’s Health Trajectories (PREDICT) cohort in Florida. Both cohorts enrolled from university-affiliated obstetric clinics. Women were eligible if the mother was > 18 years old, spoke and read English or Spanish (SHIP), or English (PREDICT), and planned to deliver at the study-affiliated hospital. Women were ineligible if the fetus had a known congenital anomaly or chromosomal abnormality, or if the mother had HIV, Hepatitis C, or Hepatitis B. Average gestational age at enrollment was 20.8 weeks (SD = 6.9) for SHIP and 24.6 weeks (SD = 6.3) for PREDICT. After enrollment, women completed questionnaires about demographics and health behaviors and provided obstetric, medical, and social histories. After delivery, parturition data and specimens were collected from participants. Umbilical cord tissue was collected to obtain human umbilical vein endothelial cells [HUVECs]). Maternal obstetric and infant delivery records were abstracted following delivery. This study was approved by the two sites’ respective institutional review boards. Mothers provided written informed consent for themselves and their children.

### HUVECs isolation

HUVECs were isolated following Crampton et al.’s protocol [25] with minor modifications. Fresh cuts were made on both ends of the umbilical cord, and excess blood drained. A blunted needle (21.5 Ga) was inserted into the vein on the placental end, secured with a hemostat. Using a 10mL syringe, the vein was flushed with Roswell Park Memorial Institute Medium (RPMI) prewarmed to 37°C until the buffer ran clear. The fetal end of the cord was then clamped with a hemostat, and 10mL 10mL Hank’s Balanced Salt Solution (HBSS) buffer with 0.05% collagenase (prewarmed to 37°C) was pushed into the vein until the vein was slightly distended for the entire length. The cord was gently massaged to confirm buffer distribution throughout the vein, and then incubated for 15 minutes at 37°C. After incubation, the cord was cut on the fetal end, above the hemostat, and incubation buffer drained into a 50mL centrifuge tube. The vein was rinsed with a further 10mL of RPMI, collected in the same tube. Cell pellets were collected by centrifugation at ~ 1300rpm for 10 minutes, supernatant discarded, and cells stored at −80°C.

The enzymatic digestion method described above was validated on a control umbilical cord, wherein the collagenase activity was confirmed to preferentially remove only endothelial cells, as indicated by CD31 immunohistochemistry performed on pre- and post-digestion cords (**Supplementary Fig. 6**).

Purity of recovered HUVEC cells and viability assays were performed on a subset of 10% of samples prior to freezing. Viability was determined using AOPI staining and visualization on a Nexcelom Vision Cellometer.

### DNA Extraction

DNA was isolated from HUVECs using an automated STAR liquid handler (Hamilton, Reno, NV, USA) integrated with a Chemagic MSM I Instrument (Perkin Elmer, Aachen, Germany) and the chemagic STAR DNA Blood3k Kit (Perkin Elmer Health Sciences, Shelton, CT). A subset of the final eluted DNA was assessed for purity and concentration using a Nanodrop 2000 UV Spectrophotometer (ThermoFisher Scientific, Waltham, MA, USA) and Tapestation 2200 (Agilent Technologies, Santa Clara, CA, USA).

### Preprocessing of Methylation Data

Methylation levels of 459 samples including technical replicates were measured with the Illumina MethylationEPIC array. Raw data were preprocessed and quantile normalized with the R package minfi [26]. At the probe level, the following probes were removed: those with detection p-value larger than 0.01 in more than 5% of total samples; those with SNPs at CpG sites; those on sex chromosomes; and cross-reactive probes [27]. A total of 64,254 probes were removed (801,605 probes remained). Functional normalization with control probes also was adopted for batch effect correction [28]. At the sample level, we filtered samples according to the following criteria: samples with more than 1% (8,658) low-quality probes (i.e., probes with detection p-value larger than 0.01); samples with mismatched sex between our records and prediction from methylation data; and samples with DNA contamination (log2 odds ratio of contamination larger than - 2) [29]. There were 50 samples with > 1% low-quality probes, 18 sex-mismatched samples, and 32 samples with contamination. In total, 71 samples were filtered during quality control (**Supplementary Fig. 7**). Lastly, 48 samples were technical replicates. The sample with fewer low-quality probes for each pair of the technical replicates was included. We also excluded a pair of twin infants, two newborns without gestational age. Thus, the analytic samples included 336 infants (Table 1). There were only negligible or small effect size difference of phenotype data after data cleaning [30].

### Epigenetic Clocks

Epigenetic gestational age was estimated with 5 different methods: Horvath’s pan-tissue clock [13], PedBE clock [4], Knight’s clock [6], Bohlin’s clock [7] and EPIC gestational age (GA) clock [8]. We omitted Falick Michaeli et al’s clock since it was created from RRBS rather than array technology, had a small sample size (n = 41) and the correlation between epigenetic and clinical gestational age was modest (R = 0.77).

Horvath’s clock was built from pan-tissue samples with a mean age of 43 years. The PedBE clock was created from buccal epithelial cells from samples between 0 and 20 years. The other three clocks were created specifically for newborns to estimate epigenetic gestational age at birth. Cord blood or blood spot DNA was used in Knight’s clock, while cord blood DNA was used in both Bohlin’s and EPIC GA clocks.

To create a clock based on our data, following Horvath [13], we used elastic net regression with R package glmnet [31]. We set alpha to 0.5 and lambda was selected using the cv.glmnet function in the glmnet package with 10-fold cross validation, which minimizes the mean absolute error of the model. The epigenetic gestational age calculated from our clock was compared to clinical gestational age at birth abstracted from the obstetric medical record.

### Epigenetic Gestational Age Acceleration and Associated Covariates

Epigenetic gestational age acceleration (EGAA) was calculated as the residual of linear regression of epigenetic gestational age on clinical gestational age. A positive value indicates an individual’s methylation age is older than their clinical gestational age.

### Social Adversity and Perinatal Outcomes

We also examined the association of EGAA with social adversity scores and perinatal covariates in this study. Social adversity was measured using summary scores from maternal reports of her adverse childhood experiences (ACEs) prior to age 18; experience of discrimination (Everyday Discrimination Scale); past-month perceived stress (Perceived Stress Scale); prenatal distress (Prenatal Distress Scale); financial stress (Financial Stress Index); anxiety (PROMIS Emotional Distress-Anxiety-6a); and depressive symptoms (Center for Epidemiological Studies-Depression Scale). In addition, we evaluated social support (Duke/UNC Functional Social Support Questionnaire), a potential buffer from adversity. A complete description of these measures can be found in the supplementary materials file. Perinatal covariates evaluated were study cohort (SHIP or PREDICT), neonatal health concern as indicated by transfer to the neonatal intensive care unit (yes/no), presence of a pregnancy complication (e.g., pre-eclampsia), maternally-reported infant race/ethnic identity (non-Hispanic White, non-Hispanic Black, or Hispanic), and infant birth weight (measured in grams). T-tests were used to test the association of EGAA with categorical covariates. We applied linear regression to evaluate the association between EGAA and continuous variables. Associations were considered significant if the p-value was < 0.05.

## Figures and Tables

**Table 1:** Demographic and clinical characteristics of newborns.

Variables		Mean ± SD or N (%)		
		All (n = 336)	PREDICT (n = 117)	SHIP (n = 219)
Gestational Age (weeks)		38.83 ± 1.58	39.24 ± 1.21	38.61 ± 1.71
Birth Weight (grams)		3307 ± 551	3427 ± 498	3243 ± 569
Sex	Male	189 (56.2)	66 (56.4)	123 (56.2)
	Female	147 (43.8)	51 (43.6)	96 (43.8)
Race/Ethnicity	Hispanic	83 (24.7)	8 (6.8)	75 (34.2)
	Non-Hispanic Black	130 (38.7)	14 (12.0)	116 (53.0)
	Non-Hispanic White	119 (35.4)	92 (78.6)	27 (12.3)
	Other	4 (1.2)	3 (2.6)	1 (0.5)
NICU	Yes	33 (9.8)	6 (5.1)	27 (12.3)
	No	281 (83.6)	94 (80.3)	187 (85.4)
	Unknown	22 (6.5)	17 (14.5)	5 (2.3)
Pregnancy Complications	Yes	118 (35.1)	10 (8.5)	108 (49.3)
	No	218 (64.9)	107 (91.5)	111 (50.7)

## Data Availability

The datasets used and/or analysed during the current study are available from the corresponding author on reasonable request.

## Abbreviations

HUVEC: human umbilical vein endothelial cell
NICU: neonatal intensive care unit
EGAA: epigenetic gestational age acceleration
ACEs: adverse childhood experiences
FIN: financial stress index
CES-D: 20-item Center for Epidemiological Studies-Depression
PSS: Perceived Stress Scale
PDS: Prenatal Distress Scale
SS: Duke/UNC Functional Social Support Questionnaire
ANX: PROMIS Emotional Distress-Anxiety-6a
EDS: Everyday Discrimination Scale
